# Blueberry Phenolics Reduce Gastrointestinal Infection of Patients with Cerebral Venous Thrombosis by Improving Depressant-Induced Autoimmune Disorder via miR-155-Mediated Brain-Derived Neurotrophic Factor

**DOI:** 10.3389/fphar.2017.00853

**Published:** 2017-11-27

**Authors:** Ning Xu, Hao Meng, Tianyi Liu, Yingli Feng, Yuan Qi, Donghuan Zhang, Honglei Wang

**Affiliations:** Department of Neurosurgery, The First Hospital of Jilin University, Changchun, China

**Keywords:** cerebral venous thrombosis, depression, gastrointestinal infection, brain-derived neurotrophic factor, miR-155

## Abstract

Cerebral venous thrombosis (CVT) often causes human depression, whereas depression-induced low immunity makes the patients susceptible to gastrointestinal infection. Blueberry possesses antidepressant properties which may improve autoimmunity and reduce gastrointestinal infection. Brain-derived neurotrophic factor (BDNF) performs antidepressant function and can be regulated by miR-155, which may be affected by blueberry. To explore the possible molecular mechanism, blueberry compounds were analyzed by high-performance liquid chromatography. Activity of compounds was tested by using HT22 cells. The present study tested 124 patients with CVT-induced mild-to-moderate depressive symptoms (Center for Epidemiologic Studies—Depression Scale [CES-D] ≥16) and gastrointestinal infection. Patients were randomly assigned to blueberry extract group (BG, received 10 mg blueberry extract daily) and placebo group (PG, received 10 mg placebo daily). After 3 months, depression, gastrointestinal infection and lipid profiles were investigated. Serum miR-155 and BDNF were measured using real-time quantitative polymerase chain reaction and or Western Blot. Blueberry treatment improved depressive symptoms and lipid profiles, and also reduced gastrointestinal infection in the BG group (*P* < 0.05) but those of the PG group (*P* = 1). These changes were paralleled by increase in serum levels of BDNF and miR-155 (*P* < 0.05). HPLC analysis showed that blueberry extracts were the main phenolic acids with 0.18, 0.85, 0.26, 0.72, 0.66, 0.4,1, and 1.92 mg/g of gentisic acid, chlorogenic acid, [2]-epicatechin, p-coumaric acid, benzoic acid, p-anisic acid, and quercetin in blueberry extracts, respectively. Phenolics in blueberry are possible causal agents in improving antidepressant activity and reducing gastrointestinal infection. Administration of blueberry increased BDNF expression and miR-155. Blueberry cannot affect BDNF level when miR-155 is overexpressed or inhibited. Phenolics from blueberry reduced gastrointestinal infection of patients with CVT by improving antidepressant activity via upregulation of miR-155-mediated BDNF.

## Introduction

Cerebral venous thrombosis (CVT) is an arterial disease, and it often results in cerebral infarction and even potential morbidity. Thrombosis may result in mental retardation (Lohiya et al., 2005), which is often associated with long-term depression (Niere et al., 2012). Depression is linked with some autoimmune diseases (Benros, 2016), which can increase incidence of bacterial infection (Alam et al., 2014). Epidemiological studies strongly suggest a link between depression and thrombosis (Stein et al., 2017). Mental depression may cause gastrointestinal disturbances (Ledochowski et al., 2000), whereas pathogens may increase in numbers and infect or invade tissue. The association of depressant symptoms with gastrointestinal infection remains unclear. Chronic psychological stress is assumed to be related to the diseases in which the immune activity fails to exert protecting functions. Chronic stress may also result in bacterial infection (Kiank et al., 2006).

On the other hand, gastrointestinal infection is often associated with thrombotic events (Altamirano et al., 2010). As reported in literature, infection can cause hemostatic abnormalities (Choi et al., 2004; Levi et al., 2012), which can lead to thrombotic complications or intravascular coagulation (Cummins et al., 2001; Asakura et al., 2006). Intestinal inflammation may activate coagulation (Schoots et al., 2004). Thrombin is associated with blood coagulation and its dysregulation may result in hemostatic abnormalities, which range from subtle subclinical to life-threatening coagulopathies (Danckwardt et al., 2013). Proinflammatory cytokines and immune cells also exert differential effects on the coagulation and fibrinolysis (Daubie et al., 2006; Pang et al., 2016). In some cases, some infections may cause hemorrhagic fever or thrombotic microangiopathy.

Phenolic-rich food was proven effective in antidepressant treatment (Chang et al., 2016; Sawamoto et al., 2016). Benefits of phenolics in human health have been well-known. Abundance of flavonoid-derived products in the gastrointestinal tract has been reported to contribute to health benefits of phenolics (Goto et al., 2012; Wang et al., 2015).

Phenolics and their metabolites have been found to transverse blood-brain-barrier and exhibit neuropharmacological properties (Shen et al., 2015; Lan et al., 2016). For instance, blueberry has been shown to increase brain-derived neurotrophic factor (BDNF) (Williams et al., 2008; Rendeiro et al., 2012). Considerable evidence suggests that BDNF plays a key role in antidepressant treatment (Wang et al., 2017). On the other hand, an earlier report has shown that miR-155 is a target of inflammatory mediators and associated with psychiatric and neurodevelopmental disorders (Thounaojam et al., 2014). Furthermore, miR-155 has been found to be a novel regulator of BDNF (Varendi et al., 2014). Thus, miR-155 may affect depressant situation of patients via BDNF.

Above information suggest that CVT often causes human depression, whereas depression-induced low immunity makes the patients susceptible to gastrointestinal infection. BDNF performs antidepressant function and can be regulated by miR-155. Blueberry may improve depression-induced autoimmunity disorder and reduce gastrointestinal infection by affecting BDNF level via miR-155. To explore the possible molecular mechanism, the effects of blueberry juice on the depression and gastrointestinal infection investigated here. Meanwhile, the main bioactive compounds of blueberry juice were measured here.

## Materials and methods

### Materials

Antibodies anti-glyceraldehyde 3-phosphate dehydrogenase (GAPDH), anti-BDNF and horseradish peroxidase-conjugated goat anti-rabbit secondary antibody were purchased from Abcam. ECL reagent and Hyperfilm ECL were purchased from Amersham Biosciences (Amersham, UK). Pure standards of phenolics and phenolic acid standards, gallic acid (Cat. No. 7384), gentisic acid (Cat. No. G5129), (1)-catechin (Cat. No. C0567), chlorogenic acid (Cat. No. C3878), caffeic acid (Cat. No. C0625), (2)-epicatechin (Cat. No. 855235), p-coumaric acid (Cat. No. C9008), sinapic acid (Cat. No. D7927), benzoic acid (Cat. No. B3250), p-anisic acid (Cat. No. A0631), myricetin (Cat. No. M6760), 3,4,5-trimethoxycinnamic acid (Cat. No. T70408), and quercetin (Cat. No. Q4951) were purchased from Sigma. High-performance liquid chromatography (HPLC)-grade trifluoroacetic acid and methanol were purchased from Sigma.

### Blueberry extracts

Blueberry powder was prepared as follows: fresh blueberries were purchased from local supermarkets. Then, 100 g blueberry fruits were blended by using a blender (DS-1, Shanghai. Precision Instruments Co., Ltd, Shanghai, China). The fresh juice was filtered by using a Whatman #2 (Whatman Inc.; Florham, NJ, USA) under vacuum. Clear juice was freeze-dried and powdered by using a miller. A dried powder sample (5 g) was extracted with 50 ml 80% methanol at 30°C for 1 day in a THZ-Q shaking incubator (HZQ-F, Harbin Donglian Electronic Technology Development Co., LTD., Harbin, China) at 100 r/min. The extracts were collected via centrifugation at 10,000 rpm for half an hour and filtered via a Millipore filter (0.22 μm) at room temperature.

### HPLC analysis

Standard stock solution (gallic acid, gentisic acid, [1]-catechin, chlorogenic acid, caffeic acid, [2]-epicatechin, p-coumaric acid, sinapic acid, benzoic acid, p-anisic acid, myricetin, 3,4,5-trimethoxycinnamic acid and quercetin) was prepared in methanol at concentrations of 0.2–1 mg/mL, and filtered through a membrane filter (0.45 mm). Phenolic compounds in blueberry extracts were analyzed using an reversed-phase HPLC with a Waters 600 pump controller equipped with an Eclipse XDB-C18 reversed-phase column (25 cm × 4.6 mm ID × 5 μm) (Supelco; Sigma-Aldrich Co.). Compounds were eluted with a mobile phase solvent A (ddH_2_O, pH 2.5 with trifluoroacetic acid) and solvent B (methanol). Gradient elution step started with 100% solvent A at 0 min until 50% solvent A and 50% solvent B for 40 min. Flow rate was 1.0 mL/min. The detection wavelength was set at four different UV wavelengths, 280, 320, and 370 nm because the compound mixtures have different UV spectra.

### HPLC analysis of phenolic contents in blueberry extracts

Phenolic contents in blueberry extracts were identified by using corresponding standards. Standard cures were made as follows: standard concentration was chosen based on final testing of phenolic contents in blueberry juice within the linear range. Regression equation was calculated by using peak area as Y (vertical axis), and the concentration as X (horizontal axis). Each compound was calculated correspond to the standard curve after detecting HPLC peak area and substituting corresponding standard curve equation.

### Inclusion criteria

The following inclusion criteria were used: (1) patients diagnosed with CVT based mainly on clinical manifestations and neuroimaging method; (2) diagnosis of hypertension and or chronic hypertension; (3)depressive symptoms measured with a total score ≥ 16 on the 20-item Center for Epidemiologic Studies—Depression Scale (CES-D) (Macedo et al., 2017; Siddaway et al., 2017); (4) *Clostridium difficile, Campylobacter* spp., *Aeromonas* spp., *Plesiomonas shigelloides, and Shigella* spp., and mixture organisms isolated from gastrointestinal tract tissue or drainage fluid; and (5) endoscopic findings indicating infection.

### Exclusion criteria

The following exclusion criteria were used: (1) current psychiatric symptoms that necessitated treatment as determined by clinical interview; (2) presence of a major medical problem; (3) undergoing medication that can affect gastrointestinal infection, CVT, and depression symptoms; (4) current involvement in weight loss or psychotherapy; and (5) pregnancy.

### Patients grouping

**Figure 1:**
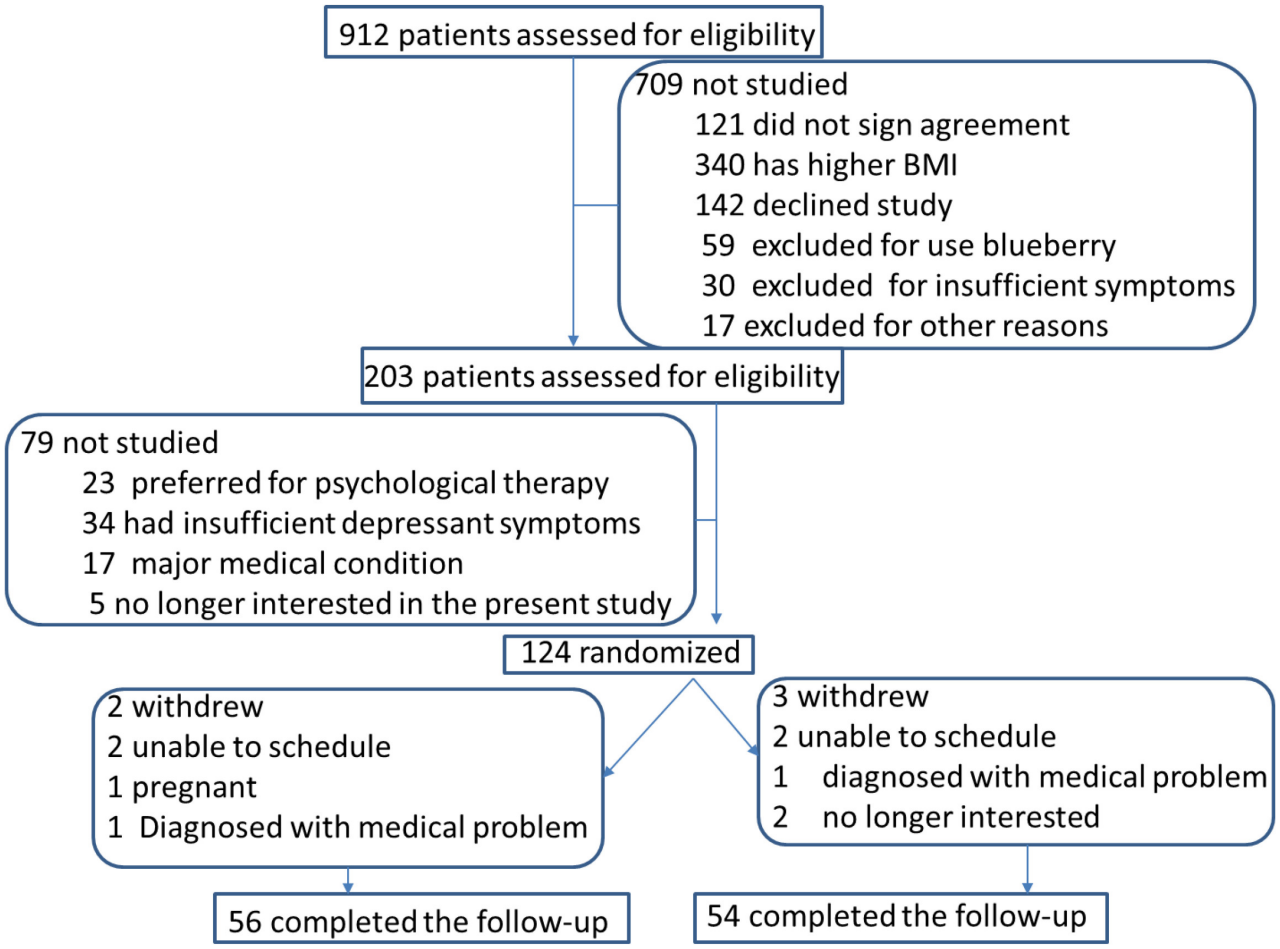
The flowchart of the present study.

Before experiments, all procedures were approved by the ethical committee of the First Hospital of Jilin University. Informed consent was obtained in writing from all patients. A total of 124 patients were selected after excluding the patients did not pass selection criteria. Final selected patients were randomly and evenly assigned into two groups: blueberry extract group (BG, *n* = 62, received 10 mg of blueberry extract daily) and placebo group (PG, *n* = 62, received placebo daily, Figure 1). Follow-up period was 6 weeks.

### Measurement of depression symptoms

Depression symptoms were measured by a total score ≥ 16 on the 20-item CES-D. Effects of blueberry on depressant symptoms were measured between BG and PG groups. Severity of depression symptoms of CVT patients were compared by using CES-D scores.

### Lipid profile measurement

Serum lipid profile (triglycerides (TG), total cholesterol (TC), low-density lipoprotein cholesterol (LDL-C), and high-density lipoprotein cholesterol (HDL-C)), which is closely associated with CVT progression, was measured. Serum concentration of TG was measured by immunometric assay (Immulite 2000 Thyroglobulin; Diagnostic Products, Los Angeles, CA, USA). Serum concentration of TC was measured by using an automated clinical chemistry analyzer kit (Biosino Biotech, Beijing, China). HDL-C was measured by using an automated chemistry analyzer (Hitachi, Tokyo, Japan). Serum concentration of LDL-C was measured by using a LDL-cholesterol kit (Sekisui Medical Co., Ltd., Tokyo, Japan). Serum malondialdehyde (MDA) was analyzed by using a MDA kit in Nanjing Jiancheng Institute of Biotechnology (Nanjing, China).

### CVT measurement

Venous thrombi were detected with computed tomography (CT) venography. Brain CT was carried out in a CT 64-slice multi-detector row CT scan (Toshiba Medical Systems Corporation, Otawara, Tochigi, Japan). CT images were obtained with 2.5 mm thickness via the posterior fossa with 5 mm section thickness via supratentorial hemispheres.

### Measurement of gastrointestinal infection

Gastrointestinal infection was first observed by endoscopy. After determination, stool specimens were received by our laboratory and were submitted with orders by providers of stool culture. Genome from the specimen aliquots was extracted by using Genomic DNA purification kit (Promega, Madison, WI, USA) according to manufacture's instruction. Bacterial species were identified by using rRNA gene bidirectional sequencing.

### Western blot

Protein was extracted by using Protein Extraction Kit (Millipore, Bedford, MD, USA) according to manufacturer's protocol. Protein concentration was determined by using a BCA protein concentration determination Kit (Beyotime, Beijing, China). Samples were incubated for 10 min at 95°C in boiling buffer (50 mM Tris, pH 8.8, 1% sodium dodecyl sulfate (SDS); 5% 2-mercaptoethanol, 10% glycerol, and 0.002% bromophenol blue). Protein samples were run on12% SDS- polyacrylamide gel electrophoresis (PAGE) and then transferred to nitrocellulose membranes. Membranes were then blocked with 5% nonfat milk (w/v) and incubated with rabbit anti-human anti-BDNF (1:1,000) or anti-GAPDH (1:3,000); and a goat anti-rabbit secondary antibody. Bands were analyzed with software Image J. 1.42q (National Institutes of Health, United States). GAPDH was used to normalize BDNF protein levels.

### Cell culture

HT22 hippocampal cells were purchased from American Type Culture Collection ATCC and were cultured in Dulbecco's Modified Eagle Medium (DMEM) with 10% fetal bovine serum (FBS), 100 μg/ml streptomycin, and 100 μg/ml penicillin in 5% CO_2_ at 37°C. HT22 cells were transfected with miR-155 mimics and inhibitors (synthesized by Shanghai Sangon Biological Engineering Technology & Services Co., Ltd., Shanghai, China) by using Lipofectamine (Thermofisher, MA, USA). Cells were treated with 200 μg/ml filtered and sterilized blueberry juice.

### Statistical analysis

For immunoblot data, statistical comparisons were carried out using to one-way ANOVA with the diet group as the main factor. Correlation coefficients were calculated using Pearson product-moment correlation coefficient. All the data were expressed as mean ±standard deviation (S.D.) and analyzed using SPSS version 20.0 (SPSS Inc., Chicago, IL, USA).

## Results

### Blueberry juice is rich in phenolics

**Figure 2:**
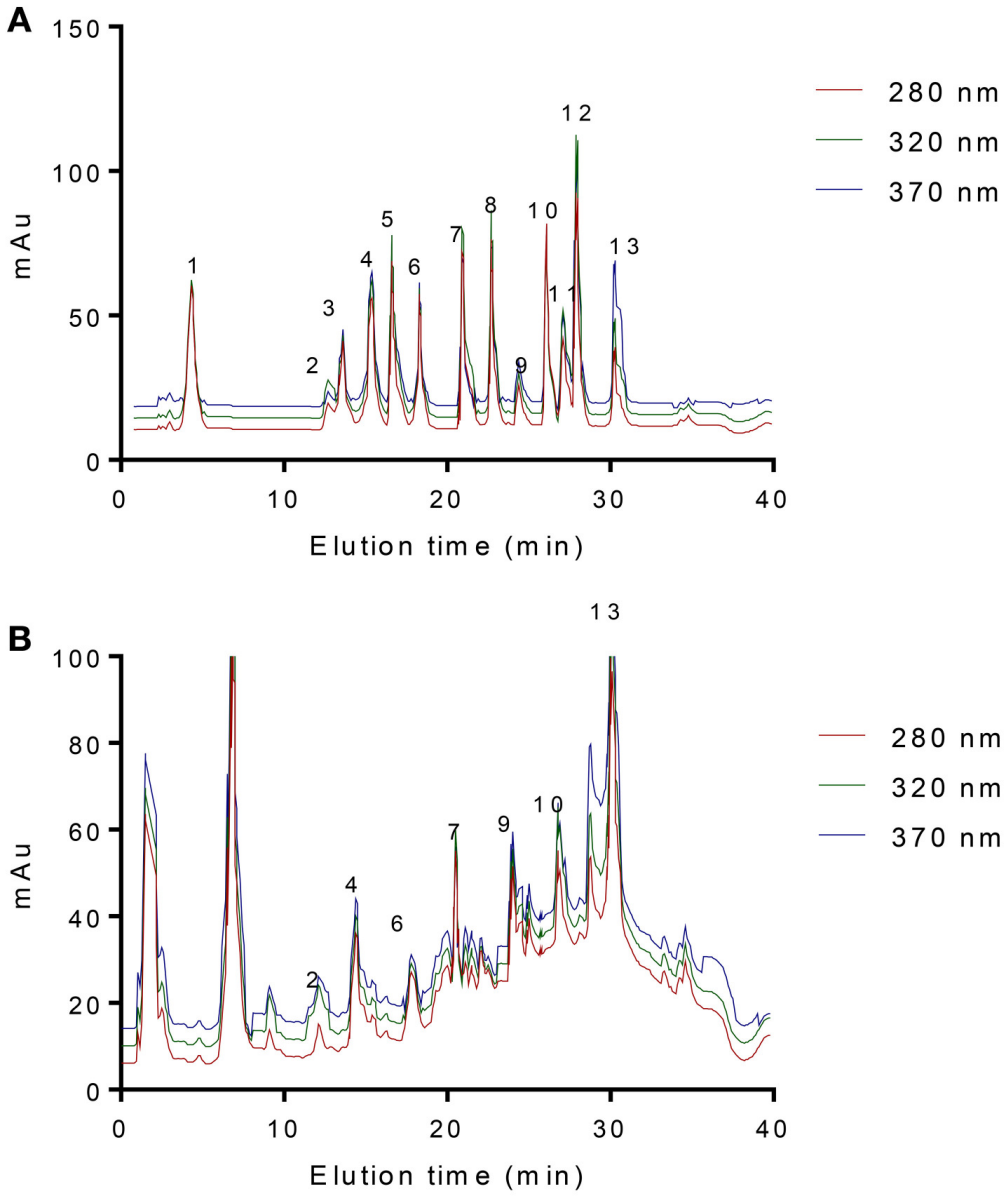
HPLC analysis of blueberry compounds. **(A)** phenolic acid standards mixture, 1 gallic acid, 2 gentisic acid, 3 (1)-catechin, 4 chlorogenic acid, 5 caffeic acid, 6 (2)-epicatechin, 7 p-coumaric acid, 8 sinapic acid, 9 benzoic acid, 10 p-anisic acid, 11 myricetin, 12 3,4,5-trimethoxycinnamic acid and 13 quercetin. **(B)** compounds from blueberry juice. 2 gentisic acid, 4 chlorogenic acid, 6 (2)-epicatechin, 7 p-coumaric acid, 9 benzoic acid, 10 p-anisic acid and 13 quercetin. The detection wavelength was set at three different UV wavelengths, 280, 320, and 370 nm.

For HPLC analysis, the maximum absorption of phenolic compounds is at 280 nm for gallic acid, [1]-catechin, [2]-epicatechin, benzoic acid and p-anisic acid; 320 nm for p-coumaric acid, gentisic acid, chlorogenic acid, sinapic acid and 3,4,5-trimethoxycinnamic acid; and 370 nm for myricetin and quercetin (Figure 2A). HPLC analysis showed that blueberry extracts are the main phenolic acids (Figure 2B, gentisic acid, chlorogenic acid, [2]-epicatechin, p-coumaric acid, benzoic acid, p-anisic acid, and quercetin) compared with standard (Figure 2A). Table 1 showed 7 kinds of phenolic compounds detected by HPLC via standards. Detection response values of standard solution had a good linear relationship between phenolic concentration and HPLC peak area, and the limit of detection are low, suggesting that this method is very sensitive. There are 0.18, 0.85, 0.26, 0.72, 0.66, 0.41, and 1.92 mg/g of gentisic acid, chlorogenic acid, [2]-epicatechin, p-coumaric acid, benzoic acid, p-anisic acid, and quercetin in blueberry extracts, respectively (Table 1).

**Table 1:** HPLC analysis of phenolic contents in blueberry extracts.

**Phenolics**	**Regression equation**	**r**	**Linear range (mg/L)**	**RSD/% (*n* = 3)**	**LOD (mg/L)**	**Blueberry extracts (mg/g)**
Gentisic acid	y = 8,435x + 9.9639	0.999 8	2.58~42.6	1.732	0.420	0.18
Chlorogenic acid	y = 23,250x − 27.688	0.999 4	3.26~ 37.4	2.20	0.48	0.85
(2)-epicatechin	y = 25,734x + 0.622	0.999 3	2.43~41.5	3.22	0.35	0.26
p-coumaric acid	y = 63,528x − 55.559	0.999 9	3.21~52.5	1.55	0.61	0.72
Benzoic acid	y = 12,723x − 6.7917	0.999 6	1.56~52.0	3.56	0.26	0.66
p-anisic acid	y = 58,236x − 41.969	0.999 2	2.70~46.0	1.28	0.50	0.41
Quercetin	y = 69,496x − 45.792	0.998 7	1.40~47.5	0.42	0.23	1.92

*r, correlation coefficient; RSD, relative standard deviation (RSD); and LOD, limit of detection. Regression equation was calculated by using peak area as Y (vertical axis), and the concentration as X (horizontal axis). Each compound was measured according to the standard curve by substituting corresponding standard curve equation*.

### Baseline clinical characteristics

Table 2 showed baseline clinical characteristics among all CVT patients with gastrointestinal infection. No statistically significant difference was observed in body mass index (BMI), daily food intake, baseline depressive symptom severity, and demographic characteristics between BG and PG groups (*P* > 0.05).

**Table 2:** The baseline clinical characters of two groups.

**Characteristic**	**BG (*n* = 62)**	**PG (*n* = 62)**	***P*-values**
**DEMOGRAPHIC**			
Age (years)	33.2 (11.6)	34.8 (10.9)	0.24
Male/female	26/36	27/35	0.86
**RACES**			
Han	57	56	0.72
Man	4	5	1.00
Meng	1	1	1.00
**ANTHROPOMETRIC**			
BMI (kg/m2)	32.7 (2.8)	31.3 (1.9)	0.14
Body fat,%	43.4 (5.7)	44.8 (6.8)	0.23
**DEPRESSIVE SYMPTOMS**			
CES-D score	36.58 (6.54)	37.29 (7.13)	0.24
CES-D score > 36, n (%)	39 (63)	38 (61)	0.85
Gastrointestinal infection	62	62	1
**CALORIE INTAKE**			
**Eating, fitness, and cortisol**			
Lunch meal intake (kcal)	1468 (170)	1506 (162)	0.22
Snack intake (kcal)	397 (43)	386 (37)	0.37
Walk/run distance (m)	1242 (298)	1164 (264)	0.39
Peak cortisol reactivity (ng/dL)	83.2 (7.5)	82.6 (9.0)	0.26

*T-Test or Chi-Square test was used to calculate the significant differences between two groups. BMI, body mass index, CES-D Center for Epidemiological Studies—Depression Scale*.

### Blueberry improves depressive symptoms of CVT patients

**Figure 3:**
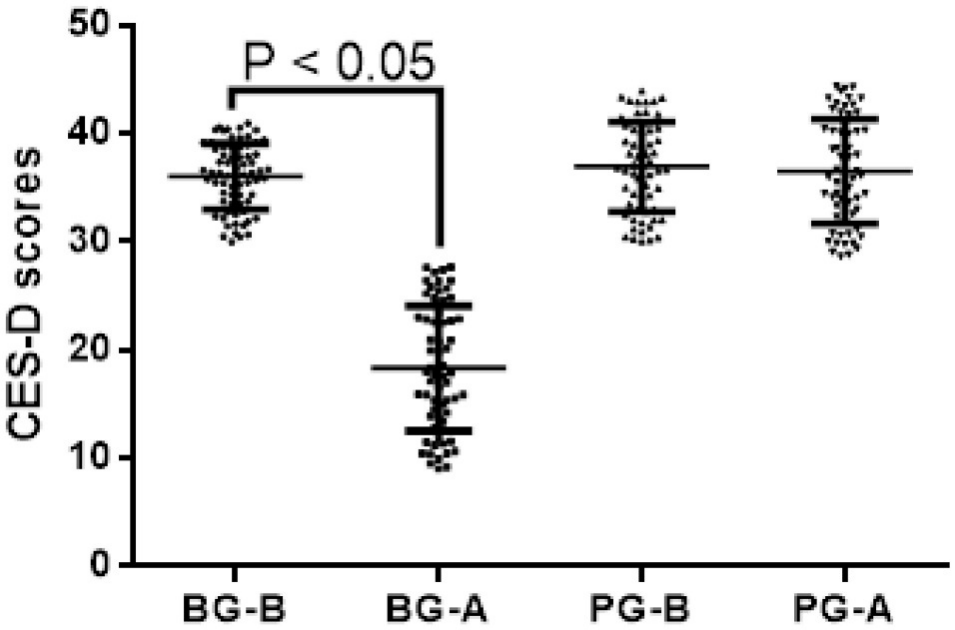
Analysis of Center for Epidemiological Studies Depression Scale (CES-D) for depressive symptoms in CVT patients between BG and PG groups. Higher scores suggest severe depressant symptoms. Cutoff point: the scores are more than 24 points are regarded as depressant patients. BG-B, CES-D scores in CTV patients from BG group before therapy; BG-A, CES-D scores in CTV patients from BG group after therapy; PG-B, CES-D scores in CTV patients from PG group before therapy; PG-A, CES-D scores in CTV patients from PG group after therapy. There is significantly statistical difference if *P* < 0.05.

As shown in Figure 3, all outcomes improved significantly between baseline and subsequent follow-ups in the BG group (*P* < 0.05) but the PG group (*P* > 0.05). Improvements from baseline to 3 months included the following: CES-D from mean (S.D.) of 36.58 (6.54) to 18.76 (9.28) (*P* < 0.001) in the BG group. Comparatively, the PG group showed no improvement from baseline to 3 months: CES-D from mean (S.D.) of 37.29 (7.13) to 36.54 (7.98) (*P* > 0.05). All patients did not use antidepressants or psychotherapy during these 3 months. After 3-month therapy, CES-D scores for depressant symptoms were reduced 48.7% when compared with before therapy in the BG group. Comparatively, there was no change for the CES-D scores for depressant symptoms in the PG group.

### CT venography analysis of CVT patients

**Figure 4:**
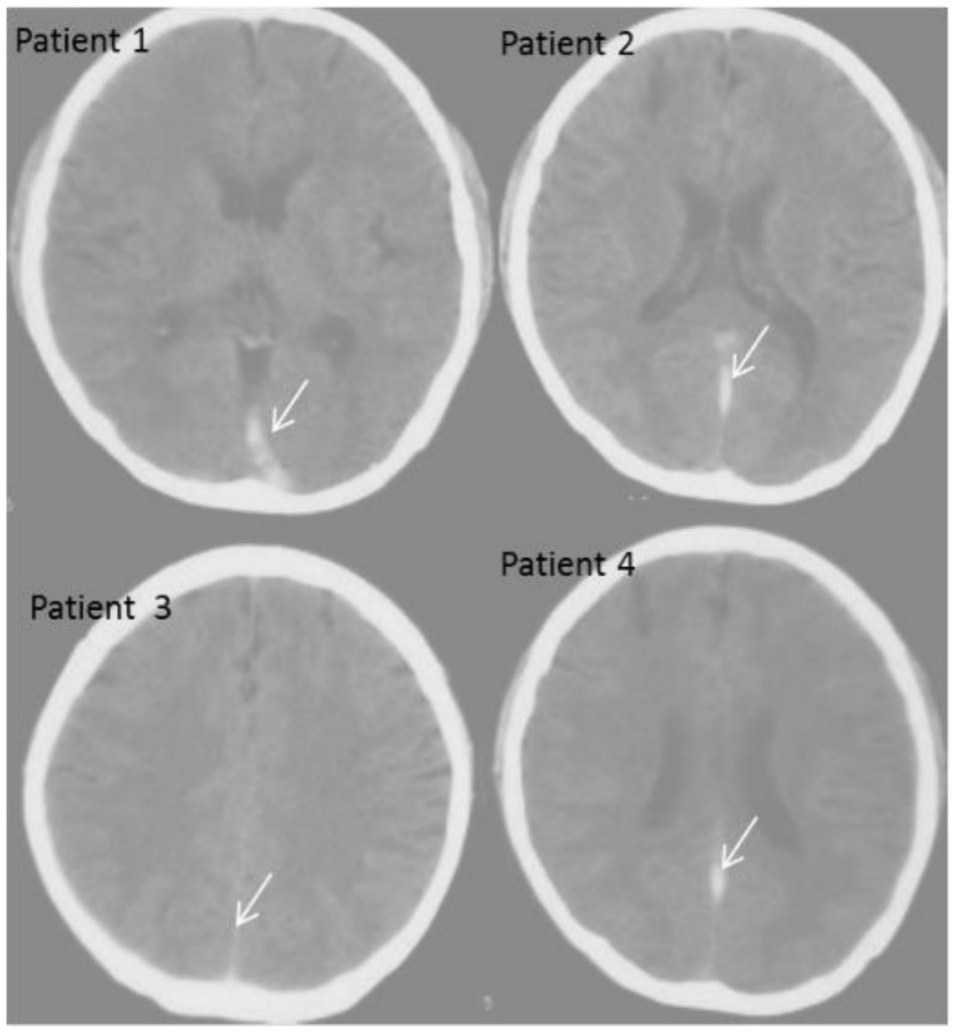
CT diagnosis of the patients with CVT. Patient 1, a male patient aged 36; Patient 2, a female patient aged 40; Patient 3, a male patient aged 34; Patient 4, a female patient age 35.

CT venography is a rapid, simple, and accurate technique for detecting CVT. The technique provides detailed information for CVT measurement. All patients were diagnosed with CVT as indicated by arrows in Figure 4. One disadvantage of CT venography is difficulty in reconstructing maximum intensity projection image from image data, which require the subtraction of bone adjacent to the venous sinus.

### Blueberry reduces the cases of gastrointestinal infection

Before therapy, no statistically significant difference was noted between BG and PG groups (*P* > 0.05). Gastrointestinal symptoms were mainly caused by infection with *Clostridium difficile, Campylobacter* spp., *Aeromonas* spp., *Plesiomonas shigelloides*, and *Shigella* spp., and mixture infection. After 3 months of blueberry therapy, the BG group presented lower cases of gastrointestinal infection than the PG group (*P* < 0.05, Table 3). After 1-month therapy, gastrointestinal infection rates were reduced by 33.9% in the BG group while the rates were only reduced by 8.1% in the PG group. After 2-month therapy, gastrointestinal infection rates were reduced by 74.2% in the BG group while the rates were reduced by 16.1% in the PG group. After 3-month therapy, gastrointestinal infection rates were reduced by 83.9% in the BG group while the rates were reduced by 21.0% in the PG group.

**Table 3:** The effects of blueberry on gastrointestinal pathogen infection.

**Pathogens**	**Before therapy**		**After 1-month therapy**		**After 2-month therapy**		**After 3-month therapy**	
	**BG**	**PG**	**BG**	**PG**	**BG**	**PG**	**BG**	**PG**
*Clostridium difficile*	10	8	5	7	4	7	3	8
*Campylobacter* spp.	5	6	3	5	3	6	2	5
*Aeromonas* spp.	5	4	2	5	2	5	0	5
*Plesiomonas shigelloides*	4	5	2	5	1	6	1	5
*Shigella* spp.	3	2	3	2	2	4	1	3
*Cryptosporidium* spp.	2	3	1	3	0	4	0	2
*Giardia* spp.	2	3	1	2	0	3	0	4
*Entamoeba histolytica*	2	2	1	1	1	3	1	3
*Salmonella* spp.	2	2	1	2	1	2	1	2
*E. coli* O157	2	2	0	2	0	3	0	2
*Yersinia enterocolitica*	1	2	1	1	0	3	0	2
Mixed infection	5	8	2	5	2	6	1	8
Total cases	62	62	41	57	16	52	10	49

### Blueberry fails to inhibit bacterial pathogens

Blueberry may inhibit gastrointestinal infection via inhibition of pathogens. To explore this possibility, the effects of blueberry on the growth of pathogens isolated from CVT patients were measured here. However, blueberry failed to inhibit the growth of bacterial pathogens even after using 10 mg/ml blueberry juice was used. Thus, blueberry extract cannot inhibit pathogens *Clostridium difficile, Campylobacter* spp., *Aeromonas* spp., *Plesiomonas shigelloides*, and *Shigella* spp., and mixture infection directly even high concentration was used (data were not shown). However, gastrointestinal infection can be inhibited by blueberry via improvement of autoimmune activity.

### Blueberry improves lipid profiles of CVT patients

Long-term blueberry consumption improved lipid profile in CVT patients from the BG group when compared with PG group. In the BG group, serum levels of TG, TC, LDL-C, and MDA decreased, whereas serum level of HDL-C changed after 1 month (Table 4). However, no statistically significant difference between the two groups (P > 0.05, Table 4). Serum lipid profile improved further in the BG group after 2 months, and statistically significant differences were observed between the two groups (*P* < 0.05, Table 4). Furthermore, the lipid profile remarkably improved in the BG group after 3 months, and the two groups featured statistically significant differences (*P* < 0.05, Table 4). These findings demonstrate that blueberry improved lipid profile in CVT patients. Therefore, blueberry treatment changed the serum levels of lipid profiles in CVT patients. Blueberry dietary supplementation not only performs antidepressant-like function but also improves lipid profile of CVT patients. Present findings demonstrate that blueberry treatment exerts antidepressant effect and regulates lipid profile simultaneously.

**Table 4:** Comparison of lipid profile in CVT patients between BG and PG groups.

		**TG(mmol/L)**	**TC(mmol/L)**	**HDL-C(mmol/L)**	**LDL-C (mmol/L)**	**MDA (mmol/L)**
Before	BG	2.76 ± 1.21	5.64 ± 1.09	1.35 ± 0.26	3.87 ± 0.94	1.67 ± 0.24
	PG	2.68 ± 0.93	5.47 ± 0.91	1.26 ± 0.35	4.16 ± 1.23	1.59 ± 0.27
	*P* value	0.62	0.73	0.46	0.52	0.43
1-month	BG	2.56 ± 1.21	5.45 ± 1.26	1.42 ± 0.20	3.45 ± 1.04	1.45 ± 0.32
	PG	2.47 ± 1.13	5.16 ± 1.18	1.33 ± 0.23	3.39 ± 0.09	1.37 ± 0.38
	*P* value	0.37	0.18	0.23	0.31	0.11
2-month	BG	2.12 ± 1.16	5.01 ± 1.06	1.56 ± 0.24	3.11 ± 1.02	1.23 ± 0.21
	PG	2.78 ± 1.34	5.81 ± 1.19	1.33 ± 0.20	4.09 ± 1.31	1.83 ± 0.25
	*P* value	0.01^*^	0.02^*^	0.04^*^	0.02^*^	0.02^*^
3-month	BG	1.75 ± 0.16	4.56 ± 1.07	1.73 ± 0.45	3.08 ± 1.13	0.90 ± 0.15
	PG	2.56 ± 0.33	5.44 ± 1.10	1.23 ± 0.18	3.92 ± 0.37	1.76 ± 0.24
	*P* value	0.01^*^	0.02^*^	0.01^*^	0.01^*^	0.01^*^

* *P < 0.05 via PG*.

### Blueberry increases BDNF level via miR-155

**Figure 5:**
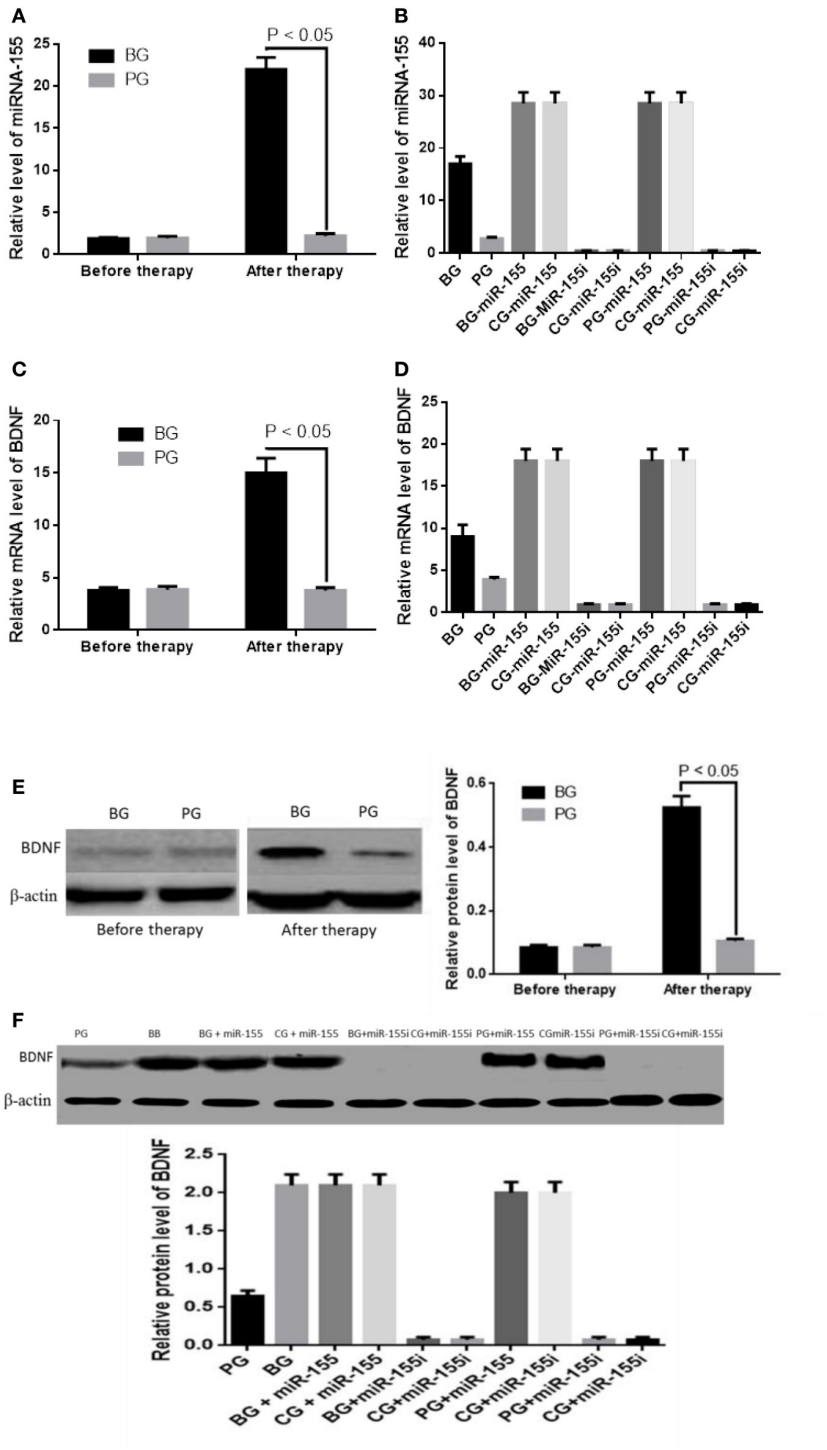
Blueberry increases BDNF level via miR-155. **(A)** Real-time qRT-PCR analysis showed the serummiR-155 levels in BG and PG groups. **(B)** Real-time qRT-PCR analysis showed the serum miR-155 levels in HT22 hippocampus cells. **(C)** Real-time qRT-PCR analysis showed relative mRNA levels of BDNF in BG and PG groups. **(D)** Real-time qRT-PCR analysis showed relative mRNA levels of BDNF in HT22 hippocampal cells. **(E)** Western Blot analysis showed the BDNF protein levels in BG and PG groups. **(F)** Western Blot analysis showed the effects of miR-155 levels on BDNF protein levels.

Real-time quantitative polymerase chain reaction (qRT-PCR) analysis showed t no statistically significant difference for serum miR-155 levels between BG and PG groups before therapy (*P* > 0.05). After 3 months of blueberry therapy, miR-155 level was higher in the BG than PG group (*P* < 0.05, Figure 5A). Relative level of miR-155 was lowest level when it was silenced whereas the level was highest when it was overexpressed (Figure 5B). Meanwhile, no statistically significant difference in BDNF mRNA levels between BG and PG groups before the therapy (*P* > 0.05, Figures 5C,E). After 3-month blueberry therapy, the BG group exhibited higher BDNF mRNA levels than the PG group (*P* < 0.05, Figure 5C). Cellular level test showed that miR-155 overexpression or inhibition can increase or reduce mRNA levels of BDNF (Figure 5D). A significant positive correlation was observed between miR-155 or BDNF levels and blueberry treatment (*P* < 0.05, Figure 5D). Blueberry treatment cannot affect the mRNA levels of BDNF when miR-155 was overexpressed or inhibited (*P* > 0.05, Figure 5D). Western Blot analysis showed no statistically significant difference in BDNF levels between BG and PG groups before the therapy (*P* > 0.05, Figure 5E). After 3 months of blueberry therapy, BDNF level was higher in BG than the PG group (*P* < 0.05, Figures 5E).Additionally, miR-155 overexpression or inhibition increased or reduced protein levels of BDNF (*P* > 0.05, Figures 5F). Blueberry treatment cannot affect t protein levels of BDNF when miR-155 was overexpressed or inhibited. All these results suggest that blueberry increases BDNF level via miR-155.

## Discussion

Blueberry has been reported to be highly effective in controlling inflammatory conditions (Johnson et al., 2017). However, the effects of blueberry on gastrointestinal infection and related molecular mechanisms remain unknown. Consumption of blueberries has been reported to improve spatial learning performance in an animal model, and this improvement is associated with the activation of BDNF pathway in the hippocampus (Rendeiro et al., 2012). Blueberry is rich in phenolics and may be useful for antidepressant treatment. Phenolic-rich food has been reported to increase the levels of BDNF, which mediates neuroplasticity, contributing critically in antidepressant treatment (Bouckaert et al., 2016). However, whether phenolics are the causal agents in inducing such behavioral responses still requires proof. We hypothesized that blueberry extracts can decrease depressive symptoms by regulating BDNF, and reducing gastrointestinal infection.

To make sure the functions of blueberry extracts, the phenolic contents should be clear. Folin-Ciocalteau method is often used to phenolic quantitation but it suffers the interference of reducing molecules other than phenolic compounds. Therefore, the phenolic quantification in blueberry extracts were performed by using HPLC analysis. HPLC analysis showed that blueberry extracts are the main phenolic acids with 0.18, 0.85, 0.26, 0.72, 0.66, 0.41, and 1.92 mg/g of gentisic acid, chlorogenic acid, [2]-epicatechin, p-coumaric acid, benzoic acid, p-anisic acid, and quercetin in blueberry extracts, respectively (Table 1).

**Figure 6:**
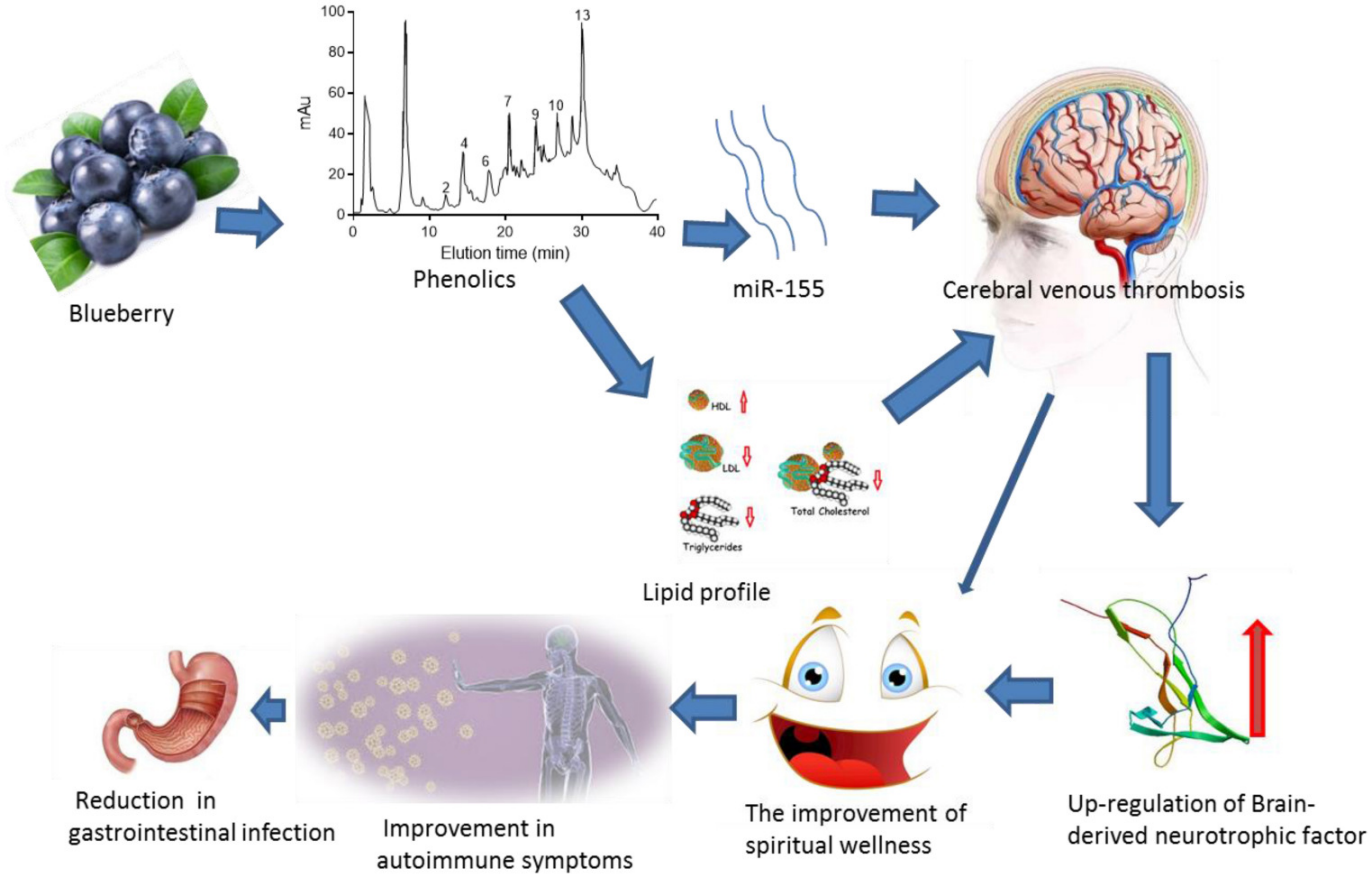
Phenolics from blueberry reduces gastrointestinal infection of the patients with cerebral venous thrombosis by improving antidepressant activity via the upregulation of miR-155-mediated brain-derived neurotrophic factor.

Blueberry polyphenols have been found to improve symptoms of neurological diseases (Dodd, 2012). Blueberries can maintain good mood of children and adults and reduce depression (Khalid et al., 2017). On the other hand, chronic depression can result in bacterial translocation or leaky gut (Maes et al., 2012). Therefore, depression may be associated with gastrointestinal infection. Antidepressant properties of blueberry may be beneficial for treatment of gastrointestinal infection. In this study, we observed that blueberry treatment can increase serum level of miR-155, resulting in increased BDNF level. BDNF can improve patients' spiritual well-being and autoimmunity, and can increase the antidepressant properties, reducing the rate of gastrointestinal infection (Figure 6).

These findings were supported by the increase in antidepressant activity induced by the phenolic-rich plants, as reported in previous reports. An interesting research discussed antioxidant properties of natural products, which can prevent brain damage from depressant disease. Enriched phenolic fraction of *C. pachystachya* exerts antidepressant function via its antioxidant activities (Ortmann et al., 2017). Antidepressant-like effect of phenolics in chronically stressed animal models was previously demonstrated by another study. Unpredictable chronic stress in an animal model also involves synthesis of pro-inflammatory cytokines, kynurenine pathway (KP), 5-hydroxytryptamine metabolism and caspase activities. Phenolics can reduce the levels or metabolism of these molecules and their activities (Filho et al., 2016). On the other hand, phenolics can prevent age-associated memory loss by scavenging free radicals and modulating BDNF levels (Souza et al., 2015). These results also suggest that the effect of phenolics in blueberry may be the main mechanism for its antidepressant function. The present data indicate that daily dose of 10 g of blueberry for 3 months can improve depressant symptoms.

According to previous reports, phenolics can inhibit pathogens, such as *Candidatropicalis, Trichophyton rubrum, Staphylococcus* species, *Escherichia coli, Clostridium* species, *Proponbacteriumacnes, Aeromonas* species, *Proteus* species, *Klebsiella* species, *Salmonella* species and *Campylobacter* species (Table 5). However, the blueberry cannot inhibit the growth of the pathogens isolated from the CVT patients with gastrointestinal infection. Therefore, blueberry inhibits gastrointestinal infection by improving autoimmune activity of CVT patients via miR-155-mediated regulation of BDNF level. On the other hand, blueberry improves lipid profiles and can inhibit CVT development. Low level of HDL-C has been found to be associated with recurrence of CVT (Ma et al., 2015).

**Table 5:** The pathogens related infection inhibited by phenolics.

**Compounds**	**Anti-bacterial activity**	**Pathogen-related infection**
7- hydroxy-3′,4′ flavone	*Candida albicans* Cushnie and Lamb, 2005	Gastrointerinal infection Remichkova et al., 2009; Glittenberg et al., 2011; Song et al., 2011
40-Methoxy flavone	*Trichophyton rubrum* Lopes et al., 2017	Skin infection Salmon and Fuller, 2013
5,7-dihydroxy-3,8-dimethoxy flavone	*Staphylococcus* species Tomás-Barberán et al., 1990; Li et al., 2014	Gastrointestinal infection Yano et al., 2000; Boyce et al., 2005
Flavone	*Escherichia coil* Li et al., 2014	Gastrointestinal infection Salyers and Whitt, 1994
2′-Hydroxyflavanone	*Clostridium difficile* Wu et al., 2014	Gastrointestinal infection Gweon et al., 2016; Khanna and Pardi, 2016; Kim et al., 2016; Furuya-Kanamori et al., 2017
5,7-dihydroxy-8-methoxyflavone	*Proponbacterium* acnes Tsai et al., 2015	Surgery infection Nodzo et al., 2014
Flavonoids	*Aeromonas* species Rattanachaikunsopon and Phumkhachorn, 2007	Gastrointestinal infection Kirov et al., 2000
Flavonoids	*Proteus* species Kumar and Pandey, 2013	Urinary tract infection Armbruster et al., 2017
Flavonoids	*Klebsiella* species Singh and Kumar, 2014	Gastrointestinal colonization Mayhall et al., 1980
Flavonoids	*Salmonella* species Abdallah and Al-Harbi, 2015	Gastrointestinal infection Nimir et al., 2009; Eguale et al., 2015
Flavonoids	*Campylobacter* species Campana et al., 2009	Gastrointestinal infection Nachamkin and Yang, 1992; Locht and Krogfelt, 2002

The main limitation of the present work is that there are many components in blueberry juice. Which component playing an important role in the therapy of CVT patients with gastrointestinal infection remains unclear. Therefore, further work is still needed to be performed to confirm the results in the future.

## Conclusions

A tight link exists between CVT and depression, whereas activation of depression results in gastrointestinal infection. miR-155 play a crucial role in mediating these effects by regulating BDNF levels. Several mechanisms contribute to enhanced risk of depression and accelerate gastrointestinal infection. Thus, blueberry treatment is possibly important in improving CVT, preventing depression symptoms, and reducing cases of gastrointestinal infection. Clinical evidence of efficacy and safety of blueberry is still needed in the future.

## Ethics statement

Before the experiments, all procedures were approved by the ethical committee of the First Hospital of Jilin University. Informed consent and assent were obtained in writing from all patients.

## Author contributions

NX and HM: conceived and designed the experiments; TL and YF: performed the experiments; YQ and DZ: analyzed the data; HM: contributed reagents, materials, analysis tools, and wrote the paper. All authors reviewed and approved this manuscript.

### Conflict of interest statement

The authors declare that the research was conducted in the absence of any commercial or financial relationships that could be construed as a potential conflict of interest.
